# Assessing Gluten-Induced Brain Fog in a Non-celiac Patient: A Case Report

**DOI:** 10.7759/cureus.93036

**Published:** 2025-09-23

**Authors:** David Oakley, Frank Palermo

**Affiliations:** 1 Medical Physics, WAVi Med, Boulder, USA; 2 Trauma, University of Colorado, Denver, USA

**Keywords:** cognitive fatigue, concussion, covid, electroencephalogram, event related potentials, gluten-induced brain fog

## Abstract

While gluten-induced brain fog is frequently reported in individuals with celiac disease, it is uncertain whether people without classic gastrointestinal symptoms of celiac may also experience similar gluten-related cognitive issues. These types of subjective complaints are difficult to quantify in clinical practice, but electroencephalography (EEG) with event-related potentials (ERPs) can be useful in identifying cognitive disturbances. The case we present here is of a 28-year-old male who reported mild cognitive symptoms associated with gluten exposure, not previously objectively evaluated. His evaluation included reaction time, trail-making, and auditory ERP assessments - modalities sensitive to conditions associated with cognition. His initial test was performed after three weeks on a normal gluten-containing diet. After this gluten exposure, the patient reported “brain fog,” which was supported by a low P300, the cognitive component of the ERP, even though he still tested strongly in reaction time and trail making. The patient was tested again after three weeks on a gluten-free diet. After eliminating gluten, the patient reported no brain fog, and this was supported by a P300 that was no longer suppressed. While this single case study cannot address causation, the ERP deficits observed in this patient were consistent with the reported “brain fog.” Objective modalities such as EEG with P300 can be readily added to the clinical assessment to aid clinicians in testing hypotheses, including situations where elimination diets are considered to address cognitive concerns.

## Introduction

Brian fog is commonly reported by patients with celiac disease (CD) when exposed to gluten, with symptoms improving with a gluten-free diet (GFD) [1]. Symptoms include a lack of mental clarity, poor concentration, and a general sense of “feeling out of it.” While research into these subtle cognitive deficits is limited, existing primarily as anecdotal reports, diffusion tensor imaging (DTI) studies have shown white matter differences between CD and control [2].

While there has been some suggestion that non-celiac gluten sensitivity (NCGS) patients can also experience subtle brain-related disorders when exposed to gluten [3,4], the question as to whether NCGS can present only as a gluten-related cognitive issue with no other classic gastrointestinal symptoms is even more open.

Whatever the case, patient complaints of diet-related brain fog need to be evaluated, and without objective tests, these cases are difficult to identify and manage, which could leave patients to suffer an otherwise readily treatable condition if, in fact, dietary changes are needed.

Patients suffering prolonged post-concussion symptoms (PPCS) and viral fatigue (such as post-COVID syndrome) often experience similar cognitive issues [5-8], and these can be measured with clear electrophysical deficits [9,10]. The clinical questions here are whether a non-CD patient complaining of brain fog with only a suspicion of a dietary link can present with similar electrophysical deficits, and if these can be changed with diet (GFD in this case).

Evoked brain responses

Electroencephalography (EEG) with auditory event-related potentials (ERP) can be an aid in the diagnosis and management of various cognitive-related issues [11,12]. EEG is the recording of the electrical signal generated by the brain through electrodes placed on the scalp, and ERPs are measurements of the EEG signal time-locked to the onset of stimuli. Patients suffering from brain fog associated with PPCS and viral fatigue can present with lower evoked-response amplitudes.

The ERP protocol used here is an auditory oddball that presents common and rare tones to the patient and then measures the brain’s response to those tones. The EEG typically records a negative voltage for both the common and rare tones at around 100 ms post-stimulus (N100), which can be used in hearing tests, where, around 300 ms (P300), a cognitive distinction that distinguishes the rare tone is made. This presents as a positive EEG voltage that is only associated with the rare stimulus. The amplitude of this P300 response is considered to be linked to the amount of cognitive resources devoted to the task of differentiating the rare tone. Larger P300 amplitudes have been associated with superior information processing [13,14], and are typically reduced in patients suffering acute concussion, PPCS, and viral fatigue [9,10,15-18].

## Case presentation

A 28-year-old male reported intermittent cognitive issues that sometimes affected his work and school over the course of many years. His complaints were subtle, though, and his slightly degraded performance was often attributed to laziness by his associates. He linked the condition not to laziness but to diet and suspected his cognition improved with a GFD even though he experienced no other classic symptoms of NCGS. This belief was never quantified, and so he sought clinical confirmation. At the patient's convenience, his first clinical visit was after three weeks of a diet with gluten (bread, pizza, and the like), where he also reported a state of “brain fog.” The second visit was after three weeks of a GFD, where he reported that the brain fog had improved.

All visits included a clinical interview, an EEG scan with a 4-min oddball audio ERP, trail making test (TMT), and reaction time test using the WAVi® system (Wavi Med, Boulder, CO) following the same procedure described elsewhere to study concussion, aging, and COVID [9,10,19,20]. TMT has been widely used in neuropsychological assessments and has been hypothesized to reflect a wide variety of cognitive processes [21,22].

Results

Table 1 shows the results of the tests, comparing the patient to expectations from an age-matched reference (control) group [20]. Here, the P300 voltage is taken from the average of the C-P locations [9,10].

**Table 1:** ERP and performance metrics in the patient pre- and post-GFD. ERP, event-related potential; GFD, gluten-free diet.

Metric	Gluten Diet	Gluten-Free Diet	Reference Range [20]
Symptoms (self-reported)	Lack of mental clarity	Improved mental clarity	
Reaction time (ms)	259	191	260-370
Trail A (ms)	37	25	37-63
Trail B (ms)	46	55	47-90
P300 voltage (μV)	7.7	12.5	7-17
P300 voltage change (μV)	-	4.6	±2

In the “brain fog” state, the P300 amplitude was at the bottom of the normal for this patient - a result also seen in the PPCS and COVID groups - suggesting a possible reduced cognitive resources even though the trail making and reaction time tests were normal. After a GFD, the symptoms resolved, and the patient’s P300 amplitude increased by 5 μV to the middle of the normal range. Given that the expected change over this time course is ±2 μV for normal patients [20], with 5 μV increases for the average PPCS and COVID patients after cognitive symptom resolution [9,10], this result is consistent with improved cognition.

**Figure 1:**
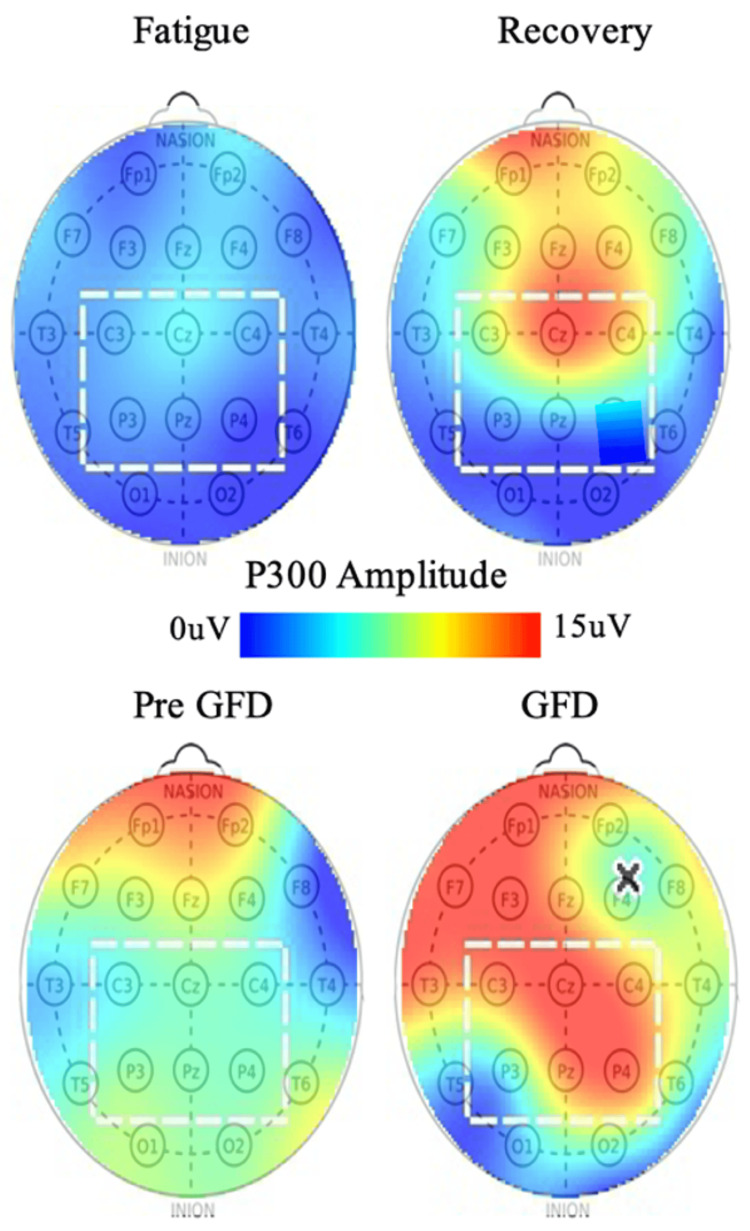
(Top) P300 amplitude as a function of scalp location for a typical post-COVID patient during the states of fatigue and recovery. (Bottom) P300 amplitude as a function of scalp location for the patient in this report pre- (with brain fog) and post-GFD (recovery). Note: The square marks the region of interest for the P300 and the "x" marks a location with poor EEG connection, outside the region of interest in this instance. GFD, gluten-free diet; EEG, electroencephalography. Top Image Source: Oakley et al., 2025 [9]. Published with permission. Open access.

The amplitude of the P300 across the scalp is shown in Figure 1 for both the GFD patient and a sample viral-fatigue patient (post-COVID) [9]. Here we find the same P300 trends for both patients where reduced amplitudes (blue-green) in the region of interest increase to red-orange with symptom resolution.

## Discussion

Brain fog is difficult to quantify clinically. While gluten-induced brain fog is frequently reported in individuals with CD [1], subjective complaints of gluten-related cognitive issues in people without classic gastrointestinal symptoms of celiac create additional diagnostic complexity.

Here, we present a non-celiac patient who reported brain fog that he associated with gluten in his diet. His suspicions were supported by EEG/ERP testing, which showed a lowered P300 amplitude that increased by 5 μV with a GFD - a similar trajectory to deficits observed in concussion and viral-fatigue recovery [9,10]. While the P300 test was sensitive to his suspected cognitive issue, the patient performed at the high end of normal on TMTA and B, and so reliance on these alone would have missed important findings. It is unclear in this case if more complex neuropsychological testing would have also detected a deficit, but the patient did not want to take on the time and expense of these tests.

This is a single case report, however, and causation cannot be established without a controlled study. Even for this patient, more data would be needed to truly address other factors and statistical variations, but these deeper evaluations are not clinically practical here. The outcome, however, is that a GFD helped the patient subjectively. While the subjective improvement may have been influenced by a placebo, the involuntary P300 result is more objective and provided the patient and clinician with information for which a continued gluten-free lifestyle was deemed appropriate. This testing modality helped the clinician generate and test a hypothesis, which is the nature of clinical practice.

## Conclusions

In this case, after three weeks on a GFD, the patient reported no brain fog. Even though no deficits were observed in cognitive testing, this symptomatic improvement was supported by EEG findings, where the cognitive P300 was no longer suppressed. While this single case study cannot address causation, the ERP deficits observed in this patient were consistent with those seen in patients suffering “brain fog” associated with prolonged concussion or viral fatigue. Objective modalities such as EEG with ERP can be readily added to the clinical assessment to aid clinicians in testing hypotheses, including situations where elimination diets are considered to address cognitive concerns.

## References

[1] Yelland GW (2017). Gluten-induced cognitive impairment ("brain fog") in coeliac disease. J Gastroenterol Hepatol.

[2] Croall ID, Sanders DS, Hadjivassiliou M, Hoggard N (2020). Cognitive deficit and white matter changes in persons with celiac disease: A population-based study. Gastroenterology.

[3] Busby E, Bold J, Fellows L, Rostami K (2018). Mood disorders and gluten: It's not all in your mind! A systematic review with meta-analysis. Nutrients.

[4] Croall ID, Hoggard N, Aziz I, Hadjivassiliou M, Sanders DS (2020). Brain fog and non-coeliac gluten sensitivity: Proof of concept brain MRI pilot study. PLoS One.

[5] Williams WH, Potter S, Ryland H (2010). Mild traumatic brain injury and postconcussion syndrome: A neuropsychological perspective. J Neurol Neurosurg Psychiatry.

[6] Voormolen DC, Haagsma JA, Polinder S (2019). Post-concussion symptoms in complicated vs. uncomplicated mild traumatic brain injury patients at three and six months post-injury: Results from the CENTER-TBI Study. J Clin Med.

[7] Graham EL, Clark JR, Orban ZS (2021). Persistent neurologic symptoms and cognitive dysfunction in non-hospitalized Covid-19 "long haulers". Ann Clin Transl Neurol.

[8] Venkataramani V, Winkler F (2022). Cognitive deficits in long Covid-19. N Engl J Med.

[9] Oakley DS, Mortazavi M, Rivera DK, Samsam L, Seitz TP, Streeter L (2025). Assessing brain neurophysiology in COVID-19 patients with prolonged cognitive fatigue: A comparison with persistent post-concussion symptoms. Cureus.

[10] Mortazavi M, Lucini FA, Joffe D, Oakley DS (2023). Electrophysiological trajectories of concussion recovery: From acute to prolonged stages in late teenagers. J Pediatr Rehabil Med.

[11] Polich J (2004). Clinical application of the P300 event-related brain potential. Phys Med Rehabil Clin N Am.

[12] Gordeev SA (2007). The use of endogenous P 300 event-related potentials of the brain for assessing cognitive functions in healthy subjects and in clinical practice. Hum Physiol.

[13] van Dinteren R, Arns M, Jongsma ML, Kessels RP (2014). P300 development across the lifespan: A systematic review and meta-analysis. PLoS One.

[14] McCarthy G, Donchin E (1981). A metric for thought: A comparison of P300 latency and reaction time. Science.

[15] Rousseff RT, Tzvetanov P, Atanassova PA, Volkov I, Hristova I (2006). Correlation between cognitive P300 changes and the grade of closed head injury. Electromyogr Clin Neurophysiol.

[16] Broglio SP, Pontifex MB, O'Connor P, Hillman CH (2009). The persistent effects of concussion on neuroelectric indices of attention. J Neurotrauma.

[17] De Beaumont L, Théoret H, Mongeon D (2009). Brain function decline in healthy retired athletes who sustained their last sports concussion in early adulthood. Brain.

[18] Clayton G, Davis N, Holliday A (2020). In-clinic event related potentials after sports concussion: A 4-year study. J Pediatr Rehabil Med.

[19] Boone J, Davids AH, Joffe D, Arese Lucini F, Oakley DS, Oakley MJ, Peterson M (2022). In-clinic measurements of vascular risk and brain activity. J Ageing Longev.

[20] Oakley DS, Fosse K, Gerwick E (2021). P300 parameters over the lifespan: Validating target ranges on an in-clinic platform [preprint]. bioRxiv.

[21] Rabin LA, Barr WB, Burton LA (2005). Assessment practices of clinical neuropsychologists in the United States and Canada: A survey of INS, NAN, and APA Division 40 members. Arch Clin Neuropsychol.

[22] Salthouse TA, Fristoe NM (1995). A process analysis of adult age effects on a computer-administered trail making test. Neuropsychology.

